# Soil Microbial Properties and Plant Growth Responses to Carbon and Water Addition in a Temperate Steppe: The Importance of Nutrient Availability

**DOI:** 10.1371/journal.pone.0035165

**Published:** 2012-04-05

**Authors:** Linna Ma, Wenwen Huang, Chengyuan Guo, Renzhong Wang, Chunwang Xiao

**Affiliations:** 1 State Key Laboratory of Vegetation and Environmental Change, Institute of Botany, The Chinese Academy of Sciences, Beijing, China; 2 Graduate School of Chinese Academy of Sciences, Beijing, China; DOE Pacific Northwest National Laboratory, United States of America

## Abstract

**Background:**

Global climatic change is generally expected to stimulate net primary production, and consequently increase soil carbon (C) input. The enhanced C input together with potentially increased precipitation may affect soil microbial processes and plant growth.

**Methodology/Principal Findings:**

To examine the effects of C and water additions on soil microbial properties and plant growth, we conducted an experiment lasting two years in a temperate steppe of northeastern China. We found that soil C and water additions significantly affected microbial properties and stimulated plant growth. Carbon addition significantly increased soil microbial biomass and activity but had a limited effect on microbial community structure. Water addition significantly increased soil microbial activity in the first year but the response to water decreased in the second year. The water-induced changes of microbial activity could be ascribed to decreased soil nitrogen (N) availability and to the shift in soil microbial community structure. However, no water effect on soil microbial activity was visible under C addition during the two years, likely because C addition alleviated nutrient limitation of soil microbes. In addition, C and water additions interacted to affect plant functional group composition. Water addition significantly increased the ratio of grass to forb biomass in C addition plots but showed only minor effects under ambient C levels. Our results suggest that soil microbial activity and plant growth are limited by nutrient (C and N) and water availability, and highlight the importance of nutrient availability in modulating the responses of soil microbes and plants to potentially increased precipitation in the temperate steppe.

**Conclusions/Significance:**

Increased soil C input and precipitation would show significant effects on soil microbial properties and plant growth in the temperate steppe. These findings will improve our understanding of the responses of soil microbes and plants to the indirect and direct climate change effects.

## Introduction

Human activity is altering the global atmosphere and climate in multiple ways. The increase in atmospheric CO_2_ concentration contributes to rising global temperatures and precipitation over some areas, including northeastern China [1], [2]. Anticipated global warming and elevated atmospheric CO_2_ concentration are generally assumed to increase primary production in most terrestrial ecosystems [3]–[5]. Consequently, much more plant residues will be incorporated into soil organic matter. Moreover, climate models predict that annual precipitation will increase by 30–100 mm in this century in the steppe [1], [6]. However, there is still a large uncertainty about how increased litter input and precipitation would affect soil microbial properties and plant growth. Some studies highlight the importance of soil microorganisms in controlling soil organic matter decomposition [7], [8]. Any changes in soil microbial activities and community structure would influence plant growth and productivity [9], [10]. The alteration of plant growth may affect soil microbial properties in return [11].

Sylvia et al. [12] and Drenovsky et al. [13] demonstrated that soil organic C availability and soil water content are particularly important factors potentially influencing soil microbial processes. Organic C availability limits soil microbial activity in most ecosystems. Thus, additions of labile organic material rapidly increased soil microbial activity and alter microbial communities by selecting for populations that are most competitive in terms of growth rates and ability to absorb nutrients [14], [15]. The relationship between soil water availability and microbial processes is complex, and usually varies with soil texture, water retention, porosity, pH and soil depth [16], [17], [18]. In semiarid ecosystems, soil microbial activity is particularly influenced by water availability. Higher water availability may increase the rates of microbial respiration and nutrient mineralization [19], [20], [21]. Alterations in soil water content will induce changes in physiology and growth of some specific groups within soil microbial communities through impacts on nutrient availability and oxygen concentrations [22], [23], [24]. In water- and nutrient-limited grassland ecosystems, increased soil organic matter and water availability would accelerate soil microbial activity and thus increase nutrient availability for plant growth and productivity [15].

Concurrent enhanced C input and increased precipitation may potentially trigger complex interactive influences on ecosystem functioning. Although several related studies have documented the combined effects of soil C and water additions on soil microbial activity and communities in farmland and stream systems [13], [25], detailed mechanistic studies evaluating their interactive effects on soil microbial properties are still limited. Available results show that regardless of organic C input, flooded soils had significantly lower ratios of fungal to bacterial biomarkers, whereas under relatively drier conditions and increased organic C availability the microbial communities had higher proportions of fungal biomass in California farmland and Australia semi-arid intermittent stream soils [13], [25]. Compared with farmland and stream systems, less is known about interactive effects of C and water additions on soil microbial properties and plant growth in temperate grassland ecosystems. Soil water, N and phosphorus (P) are key limiting factors in grasslands [26], [27], [28]. With water availability increasing, microbial activity and plant growth are expected to be more limited by nutrient availability than by water. In this context, the responses of soil microbial activity and plant growth to water addition may be stronger when C (substrate) is added.

To examine the effects of the predicted enhanced precipitation and C input in the temperate steppe of northeastern China, we conducted a field experiment in which we artificially manipulated C input to topsoils (+60%) and increased precipitation (+30%) to study the effects on soil microbial biomass, microbial activity, microbial community structure and plant growth. The specific questions addressed here were: (1) how do soil microbial properties respond to C and water additions during two growing seasons; (2) if C and water additions stimulate plant growth by the changes of soil microbial properties; and (3) whether C and water additions interact to affect soil microbial properties and plant growth.

## Results

### Soil Microclimate and Properties

**Figure 1:**
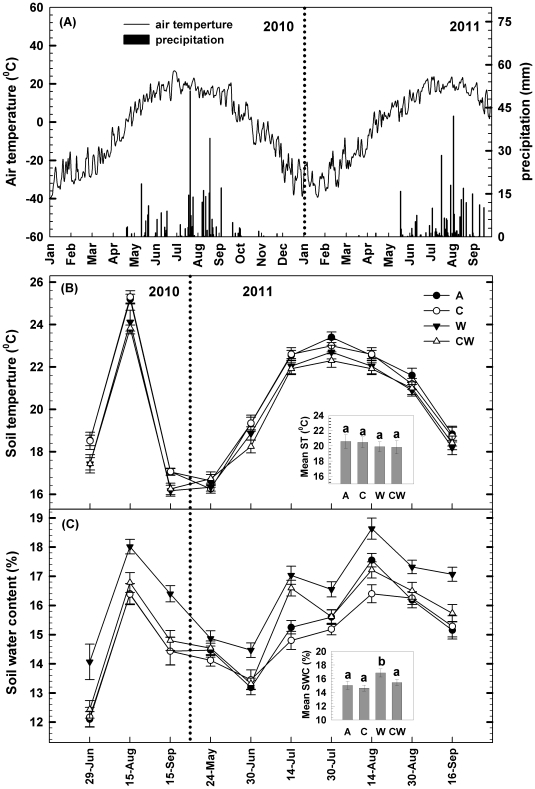
Daily precipitation (bars) and daily mean air temperature (line) in 2010 and 2011 (A). Data are from the eddy tower adjacent (approximately 100 m) to the experimental site. Seasonal variations of soil temperature (B) and water content (C) at topsoil layer (0–10 cm) in response to carbon addition (+60%) and water addition (+30%) in the temperate steppe of northeastern China. Insets represent the two seasonal mean values of soil temperature (ST) and water content (SWC). Vertical bars indicate standard errors of means (n = 6). Difference lowercase letters indicate statistically significant differences (*P*<0.05). A = ambient condition (control), C = carbon addition, W = water addition, CW = combined carbon and water additions.

Seasonal dynamics of both precipitation and air temperature exhibited one-peak patterns, which were higher in summer and lower in spring and autumn (Fig. 1A). Total precipitations over the entire growing season (May to September) in 2010 (328.8 mm) and 2011 (278.2mm) were 6% and 20.5% lower than the long-term mean (350mm), respectively. No difference in mean annual or seasonal air temperature was detected between 2010 and 2011. Soil C addition showed no effect on soil temperature and water content during the two growing seasons (Fig. 1B, C). Water addition increased soil water content by 12% and 9% (*P<*0.05, Fig. 1C) in 2010 and 2011, respectively. There was a significant interaction between soil C and water additions in affecting soil water content (*P<*0.05, Table 1, Fig. 1C), as water addition significantly increased soil water content under soil C ambient conditions but had no effect under C addition treatments.

**Table 1:** Results (*F*-values) of Four-way ANOVAs on the effects of carbon addition (C), water addition (W), sampling date (D), year (Y), and their interactions on soil temperature (ST), soil water content (SWC), microbial biomass C (MBC), microbial biomass N (MBN), microbial activity (SMA), metabolic quotient (*q*CO_2_), soil inorganic N (IN), soil total PLFAs (TP), contribution of soil fungal PLFAs (F) and bacterial PLFAs (B), and the ratio of fungal to bacterial PLFAs (F: B).

	ST	SWC	MBC	MBN	SMA	*q*CO_2_	IN	TP	F	B	F:B
C	0.18	5.94*	197.06***	102.95***	116.64***	0.34	9.55**	154.57***	2.23	3.41	3.50
W	1.95	11.72**	1.90	0.44	8.95**	3.06	67.53***	1.04	8.89**	7.27**	9.56**
C×W	0.24	7.18*	0.04	0.06	30.06***	21.80***	3.40	0.58	4.20*	4.16*	5.35*
D	41.43***	134.54***	1041.07***	359.16***	733.76***	5.46**	46.24***	683.54***	5.12*	2.81	3.73*
D×C	0.20	0.11	0.78	3.05	0.05	0.05	0.18	0.83	0.88	0.05	0.71
D×W	0.50	0.57	0.63	0.64	1.36	2.25	0.15	0.57	2.82	2.17	2.83
D×C×W	0.39	0.18	0.18	1.34	0.69	0.68	0.10	0.43	2.06	1.15	2.19
Y	1.89	7.24**	0.16	3.08	3.76*	3.81*	13.44***	0.09	3.88*	3.75*	3.95*
Y×C	0.09	0.13	0.02	0.62	0.11	0.14	9.27**	0.14	2.04	2.11	2.16
Y×W	3.01	2.43	0.26	0.11	5.16*	5.48*	10.11**	0.31	4.66*	3.15	3.74*
Y×C×W	0.01	0.06	0.06	0.04	0.20	0.36	2.33	0.16	2.05	2.74	3.22

* , **, *and* ***represent significant at P<0.05, 0.01, and 0.001, respectively.

**Figure 2:**
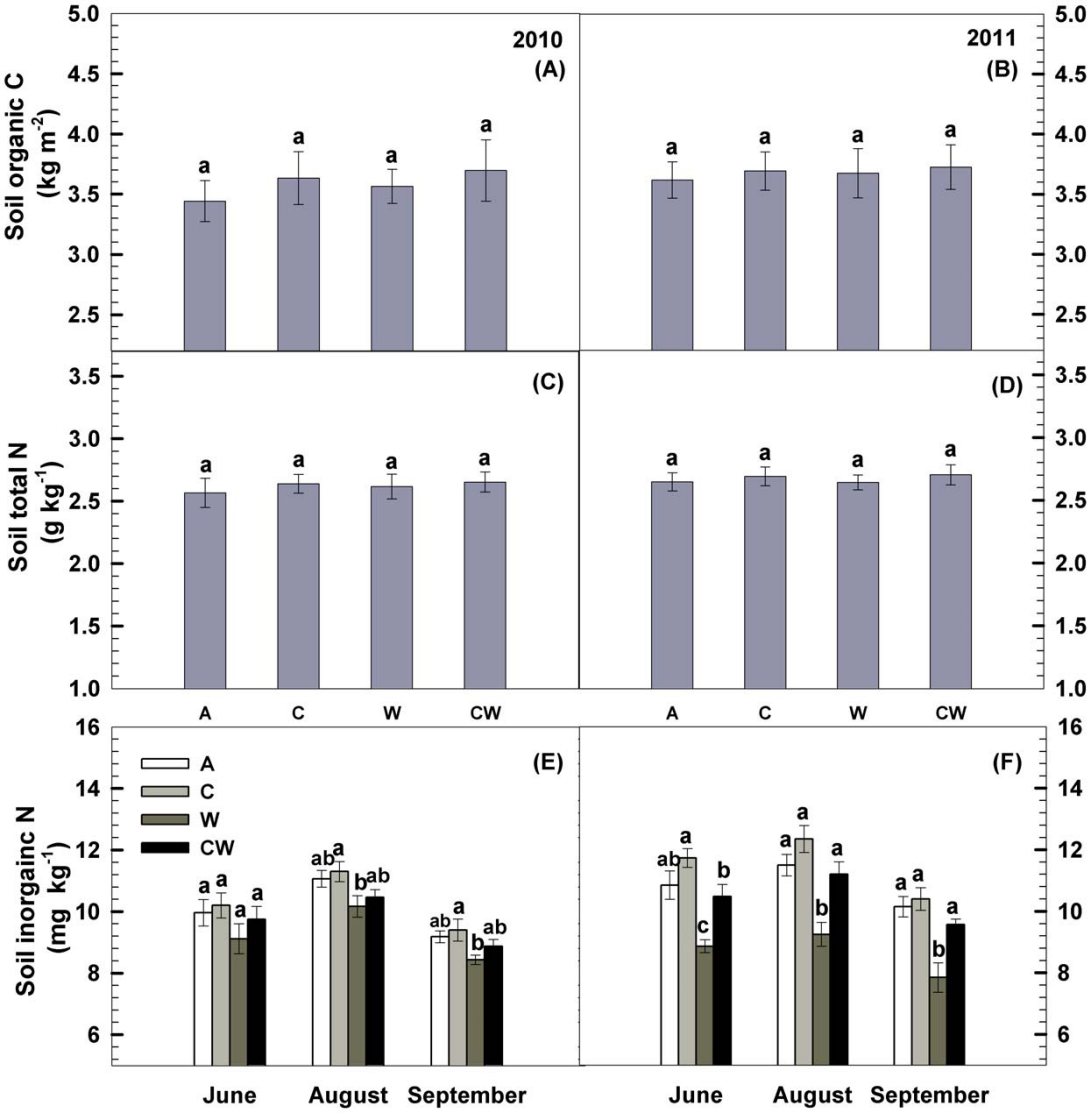
Responses of soil organic carbon (C), total nitrogen (N) and inorganic N content to carbon addition (+60%) and water addition (+30%) during the two growing seasons in temperate steppe of northeastern China. Vertical bars indicate standard errors of means (n = 6). Difference lowercase letters indicate statistically significant differences (*P*<0.05). A = ambient condition (control), C = carbon addition, W = water addition, CW = combined carbon and water additions.

Soil organic C and total N content (top 0–10 cm) were unchanged under both C and water additions during the two growing seasons (Fig. 2A-D, Table 2). Water addition caused a consistent decrease in soil inorganic N by 8.3% (*P<*0.05) and 20% (*P<*0.01, Fig. 2E, F) in the two years, respectively.

**Table 2:** Results (*F*-values) of Three-way ANOVAs on the effects of carbon addition (C), water addition (W), year (Y), and their interactions on soil organic C (SOC), soil total N (TN), aboveground biomass C (ABC) and N (ABN), root biomass C (RBC) and N (RBN), grass biomass (GB), forb biomass (FB) and the ratio of grass to forb biomass (GB: FB).

	SOC	TN	ABC	ABN	RBC	RBN	GB	FB	GB:FB
C	1.44	0.31	2.08	3.11	3.97*	6.19*	2.99	1.02	3.32
W	1.06	0.26	2.25	5.22*	1.63	4.51*	2.78	4.16*	0.69
C×W	0.64	0.18	0.53	0.77	1.34	3.64	0.57	3.52	3.94*
Y	1.75	0.64	3.04	1.53	3.72	1.04	3.89*	1.15	0.82
Y×C	0.68	0.17	0.21	0.43	5.75*	1.27	0.38	0.64	1.32
Y×W	0.92	0.32	0.36	0.71	4.38*	3.02	0.52	4.62*	0.76
Y×C×W	0.47	0.16	0.42	1.29	1.47	0.73	0.77	1.53	0.65

* *represents significant at P<0.05.*

### Soil Microbial Biomass

**Figure 3:**
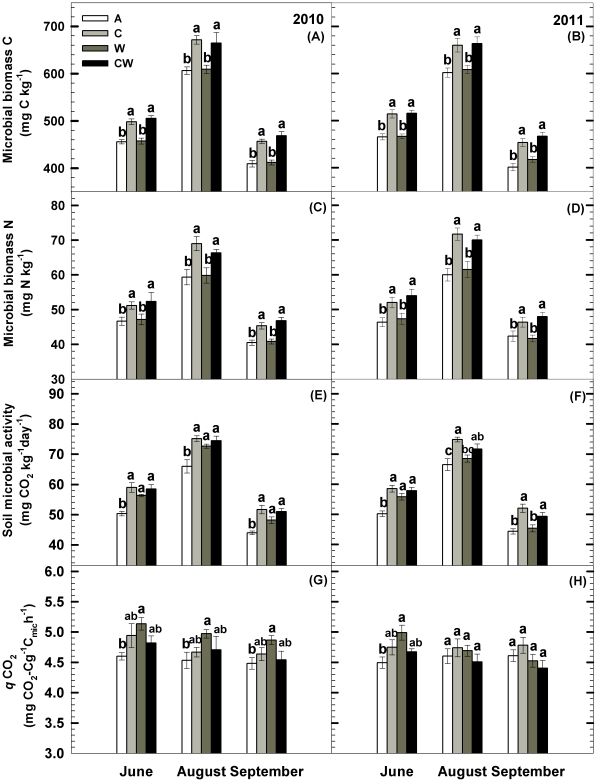
Seasonal variations of soil microbial biomass carbon (C) and nitrogen (N), soil microbial activity and microbial metabolic quotient (*q*CO_2_) in the 0 - 10 cm soil layer as influenced by carbon addition (+60%) and water addition (+30%) in temperate steppe of northeastern China. Values show the monthly means from June to September in the two growing seasons. Vertical bars indicate standard errors of means (n = 6). Difference lowercase letters indicate statistically significant differences (*P*<0.05). A = ambient condition (control), C = carbon addition, W = water addition, CW = combined carbon and water additions.

In general, both soil microbial biomass C (MBC) and microbial biomass N (MBN) showed pronounced seasonal variations with the higher values in summer and lower values in spring and autumn during the two growing seasons (Fig. 3A-D). Soil C addition increased MBC by 10.5% and 10.8% (*P<*0.001, Table 1, Fig. 3A, B), and increased MBN by 12.9% and 14.3% (*P<*0.001, Table 1, Fig. 3C, D) in 2010 and 2011, respectively. However, water addition showed no effect on MBC and MBN. Year did not interact with C (or water) addition to affect MBC and MBN. There were no significant effects of C and water additions interactions on MBC and MBN during the two growing seasons (Fig. 3A-D).

### Microbial Community Structure

**Figure 4:**
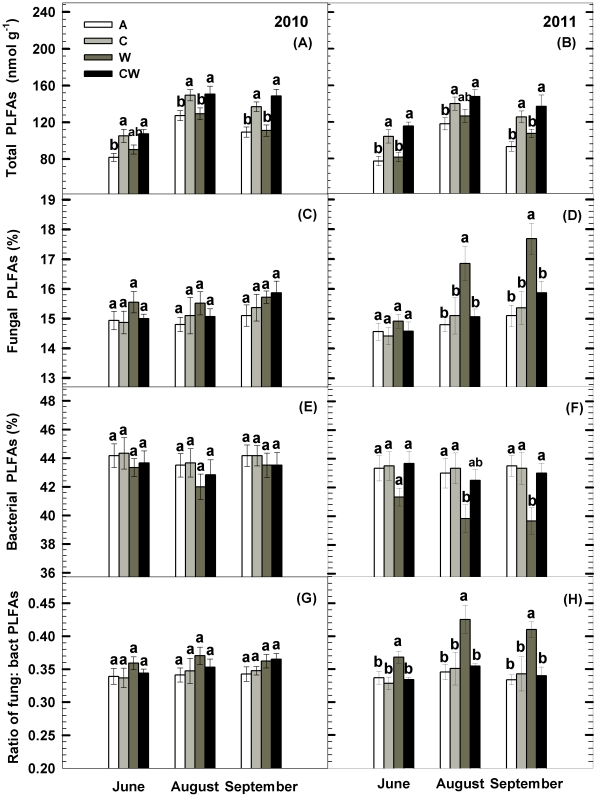
The total biomass phospholipid fatty acids (PLFAs), percentages of fungal and bacterial PLFAs to the total biomass PLFAs, and the ratio of fungal to bacterial PLFAs as influenced by carbon addition (+60%) and water addition (+30%) in temperate steppe of northeastern China. Values show the monthly means from June to September in the growing season. Vertical bars indicate standard errors of means (n = 6). Difference lowercase letters indicate statistically significant differences (*P*<0.05). A = ambient condition (control), C = carbon addition, W = water addition, CW = combined carbon and water additions.

The soil total phospholipid fatty acids (PLFAs) followed a similar pattern of seasonal dynamics to soil microbial biomass (MBC and MBN). Soil C addition increased total PLFAs by 22.8% and 28.1% (*P<*0.001, Table 1, Fig. 4A, B) in 2010 and 2011, respectively, whereas water addition had no effect. Soil C addition did not significantly affect the percentage of soil fungal PLFAs, bacterial PLFAs, and the ratio of fungal to bacteria PLFAs (F: B) during the two growing seasons (Fig. 4C-H). Water addition enhanced the proportion of soil fungal PLFAs by 11.5% and 11.8% (*P<*0.05, Fig. 4D), and reduced the proportion of bacterial PLFAs by 7.3% and 8.8% (*P<*0.05, Fig. 4F) in August and September 2011, respectively. Consequently, extra water enhanced soil F: B by 23.1% and 22.8% in August and September 2011 (*P<*0.05, Fig. 4H). There were significant interaction effects between soil C and water additions on soil F: B in August and September 2011, in that water addition significantly increased the F: B in ambient C levels but had no effect when extra C was added (Table 1, Fig. 4H). Across the 24 plots, soil F: B was negatively correlated with soil inorganic N (R = −0.50 in 2010, *P*<0.05; R = −0.69 in 2011, *P*<0.01).

### Soil Microbial Activity

Soil microbial activity (SMA) exhibited significant seasonal variations with the higher values in summer and lower values in spring and autumn (Fig. 3E, F). Soil C addition significantly increased SMA by 15.9% and 15.1% in 2010 and 2011 (*P<*0.001), whereas it showed minor effect on metabolic quotient (*q*CO_2_, Table 1, Fig. 3G, H). Water addition increased SMA by 10% (*P<*0.05) and *q*CO_2_ by 10% (*P<*0.05) in 2010 whereas it had no effect on SMA and *q*CO_2_ in 2011. There were significant effects of soil C and water additions interactions on the SMA and *q*CO_2_ (*P<*0.001, Table 1), in that the increases in SMA and *q*CO_2_ were significantly smaller than would be expected if the two factors acted additively during the two growing seasons. Across the 24 plots, SMA showed positive linear correlation with soil inorganic N (R = 0.47 in 2010, *P*<0.05; R = 0.51 in 2011, *P*<0.05).

### Plant Growth

**Figure 5:**
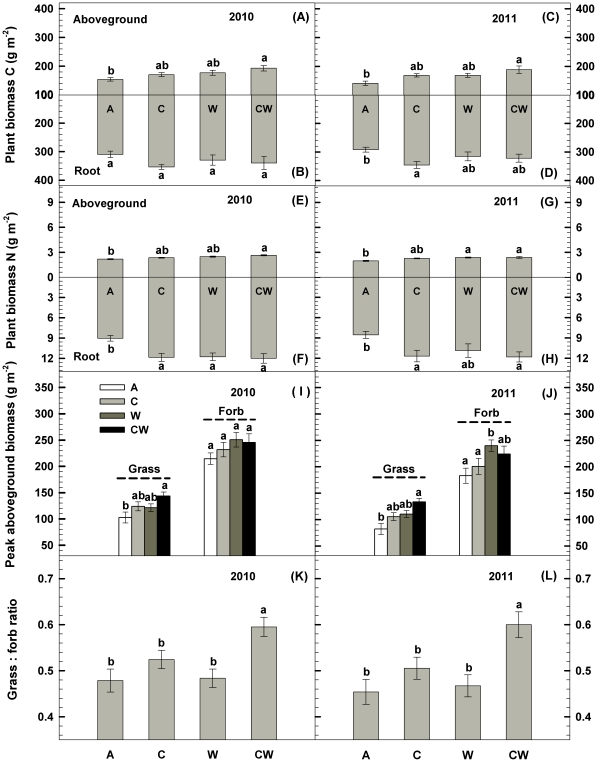
Responses of aboveground biomass carbon (C) and nitrogen (N), root biomass C and N, peak aboveground biomass of grass and forb and the grass: forb ratio to carbon addition (+60%) and water addition (+30%) in 2010 and 2011 in temperate steppe of northeastern China. Vertical bars indicate standard errors of means (n = 6). Difference lowercase letters indicate statistically significant differences (*P*<0.05). A = ambient condition (control), C = carbon addition, W = water addition, CW = combined carbon and water additions.

Soil C addition significantly increased plant root biomass C by 18.5% in 2011 (*P*<0.05, Fig. 5D) and N by 29.6% and 34.1% in 2010 and 2011, respectively (*P*<0.01, Table 2, Fig. 5F, H). Water addition significantly stimulated aboveground biomass N (13.8% in 2010, 20.3% in 2011; *P*<0.05, Fig. 5E, G), and root biomass N (27% in 2010; Table 2, *P*<0.05, Fig. 5F).

Grass and forb biomass showed differential responses to C and water additions. Carbon addition marginally increased grass biomass during the two growing seasons, whereas it did not affect forb biomass (Table 2, Fig. 5I, J). Water addition significantly increased forb biomass in 2011 (*P*<0.05, Fig. 5J). The differential responses between grass and forb biomass also induced changes of the grass: forb biomass ratio under the C and water additions. For example, water addition significantly increased the ratio of grass to forb biomass in the C addition plots but it had minor effect in ambient C conditions (Table 2, *P*<0.05, Fig. 5K, L).

## Discussion

### C Addition Effects

We found no significant changes in soil fungal to bacterial PLFAs ratios (F: B) in C addition plots during the two growing seasons, though soil C addition increased soil microbial biomass (Fig. 3A-D; Fig. 4G, H). Similarly, results from forest ecosystems showed that incorporation of forest residues into soils had no effect on soil F: B at four study sites ranging from California to South Carolina [29]. Busse et al. [29] and Morris et al. [30] found that soil F: B was relatively constant within watersheds and individual forest stands, even with large residue additions. In this temperate steppe, within-site differences of the climate and soil physical and chemical properties were minor. Thus, although a large proportion (+60%) of soil C (or substrate) was added, the soil microbial community showed modest responses. In addition, the microbial metabolic quotient (*q*CO_2_), an indicator of the C-use efficiency of the microbial community, was similar in the control and the C addition plots (Fig. 3G, H). This result compliments our finding of soil F: B and supports the apparent insensitivity of microbial community structure to extra substrate complements. Future long-term C addition experiments will clarify whether this hypothesis is valid.

In agreement with results from farmlands and forests [31], [32], [33], soil C addition greatly enhanced soil microbial activity in this temperate steppe (Fig. 3E, F). The strong response of soil organic matter decomposition to the below-ground supply of labile C indicated the energy limitation (C availability) of the microbial activity in this temperate steppe and most added C was released because soil C sequestration was relatively minor (Fig. 2A, B). Our results, therefore, suggest that enhanced soil C input in short term would accelerate soil C cycling rather than induce soil C sequestration in the temperate grasslands of northeastern China.

Given the strong response of microbial activity, the addition of C (POM) in the present study is likely to have increased nutrient availability. This may explain why C addition remarkably increased plant biomass (e.g. aboveground biomass + root biomass) (Fig. 5A-H) . This result is in line with the previous studies on effects of increased nutrient availability on plant growth in semiarid grasslands [15], [34]. These results suggest that plant productivity is nutrient (N availability)-limited in this temperate steppe of northeastern China.

### Water Addition Effects

Although water addition showed no effect on soil microbial biomass, it significantly increased the soil F:B in the second growing season (Fig. 4H), which may contribute to the observed shift of microbial community structure showing an increasing contribution of soil fungi. In water addition plots, we found increased above- and below-ground plant N (Fig. 5E-H) and decreased soil inorganic N content (Fig. 2E, F) in topsoil layer, indicating that the enhancement of plant growth may accelerate soil nutrient transfer from soil to plants and significantly reduce soil N availability. The reduced soil N availability would intensify nutrient competition between plants and soil microbes and result in nutrient limitation of microbes [35]. When soil nutrient availability is low, fungi can relocate nutrients due to their filamentous nature and recycle limited nutrients (especially inorganic N) via cytoplasm translocation. This feature may enhance fungi competitive advantages over bacteria for exploitation of available nutrients [36]. Thus, the decrease in soil N availability likely contributes to the enhancement of fungal dominance in the whole soil microbial community [37], [38].

There have been many reports showing positive responses of soil microbial activity to increased precipitation (or water addition) in arid and semiarid ecosystem [15], [39]. In contrast, we found that the effects of water addition on soil microbial activity were inconsistent during the two years (Fig. 3E, F). The water-induced changes in soil microbial activity could be ascribed to the decrease of soil N availability and the shift in the soil microbial community structure (Fig. 4H). Firstly, the decrease of soil inorganic N availability following water addition likely leads to nutrient limitation for the soil microbial activity [35], [40]. Secondly, the increase in fungal proportion may alter the decomposition process from a faster bacterial-based channel to a slower fungal-based channel [41], [42], [43] .

There was a positive response of the plant biomass (aboveground biomass + root biomass) to water addition, although no shift in plant functional groups composition was observed (Fig. 5A-H). This is similar to the results from some previous studies in the temperate grassland ecosystems subjected to increased precipitation [27], [34]. These results suggest that soil microbes and plant growth are also limited by water availability in this semiarid grassland.

### Interactive Effects of Combined C and Water Additions

Our results showed that no water effects on soil microbial activity and community structure were visible under C addition during the two years (Fig. 3E, F; Fig. 4G, H), this is likely because C input (and the associated nutrient release) alleviates energy and nutrient limitation to soil microbes. Hence, water-induced alterations in microbial activity and community structure in the second year are masked by soil C addition.

The interaction between C and water additions caused the shift in plant functional groups composition due to the increase of grass biomass (Table 2, Fig. 5I-L). That is, water addition significantly increased the ratio of grass to forb biomass in the C addition plots but it showed no effect in ambient C conditions. The positive response of grass biomass could be primarily ascribed to the enhancement of dominant species (*Stipa baicalensis* and *Leymus chinensis*) that can quickly explore available resources relative to other species [44]. These findings highlight that the multifactor effects would be more complex than simple combinations of single-factor responses. Considering the unprecedented enhancement of soil C input associated with expected changing precipitation regime changes under global climate change [45], multifactor experiments are needed to fully understand the impacts of global change on terrestrial ecosystem structure and function.

### Conclusions

With a field manipulative experiment, this study showed that enhanced soil C input and water addition affected soil microbial processes and stimulated plant growth in the temperate steppe. Soil C addition increased soil microbial biomass and activity but showed no effect on microbial community structure. Water addition increased soil microbial activity in short term and then showed minor influence on microbial activity as the water addition progresses. However, this alteration was invisible under C addition. Moreover, combined C and water additions caused the shift in plant functional groups composition due to the increase in the ratio of grass to forb biomass. Our results indicated that soil microbial activity and plant growth are limited by nutrient (e. g. C and N availability) and water availability, and that nutrient availability would regulate the effects of water availability in the temperate grassland. We conclude that soil microbial properties and plant growth would be more pronounced in response to potential future enhanced soil C input and increased precipitation. Further long-term multifactorial field experiments will be needed to capture potential effects of global changes on soil microbial processes and plant growth.

## Materials and Methods

### Ethics Statement

Hulunber Grassland Ecosystem Observation and Research Station is a department of Chinese Academy of Agriculture Sciences. This study was approved by State Key Laboratory of Vegetation and Environmental Change, Institute of Botany, the Chinese Academy of Sciences and Hulunber Restoration Ecology Experimentation and Demonstration Station.

### Study Site and Experimental Design

A typical native steppe was selected in Hulunber Grassland Ecosystem Observation and Research Station, which is located at Xiertala farm, the center of Hulunber Meadow steppe. This field site is situated at the most east part of Eurasia steppe, northeastern China (Latitude 49° 19′ N, Longitude 120° 02′ E, Altitude 628 m). Mean annual air temperature is −3 ∼ −1°C. The mean precipitation during the summer (May to September) was approximately 350 mm for periods 1980–2009, 328.8 mm and 278.2 mm in 2010 and 2011, respectively. Mean growing season length is approximately 150 days. Soils in the experimental sites are characterized as chestnut (Chinese classification; soil texture: sand is 42%, silt is 35% and clay is 23%), with low nutrient availability in the surface layer. Soil bulk density is 1.37 g cm^-3^ and pH is 7.7 (for top 10 cm). The vegetation at the site was dominated by perennial grasses (*Stipa baicalensis*, *Leymus chinensis*) and forbs (*Artemisia frigida*, *Serratula centauroides*). Total vegetation ground cover ranges from 60–75%.

The experiment was based on a complete randomized block factorial experimental design with soil C and water additions as fixed factors and two levels in each factor in mid-May of 2010. Twenty-four 2×2 m plots were established at the grassland site and plots were exposed to ambient conditions (control), soil C addition, water addition and combination of soil C and water additions. There were six replicates for each treatment.

Treatments of enhanced soil C input were addition of fresh particulate organic matter (POM) to the surface soil layer (0–10 cm) at the rate of 288 (+60%) g C m^-2^. The fresh POM used in this study consisted of senescent above-ground tissues from a mix of plant species occurring at the site. Senescent plant biomass was harvested from an adjacent field, air-dried and milled to 1–2 mm before use. The C and N contents, the C: N ratio, and the P and lignin contents of the POM were 40.33% (standard error (SE) = 2.64%; n = 6), 0.32% (SE = 0.03%; n = 6), 144.6 (SE = 13.2%; n = 6), 0.025% (SE = 0.002%; n = 6) and 20.41% (SE = 1.24%; n = 6), respectively.

We expected to add the POM to the upper soil layers without drastically damaging the root systems. For this purpose, we carefully used sharp forks to loosen the surface soil (10 cm), and a predetermined quantity of POM was gradually and homogeneously added to the soil in the 0–10 cm layer. The soil pores were carefully filled with soil and gently compacted by hand. To create consistent soil disturbance across treatments, the plots with no POM addition were processed in the same manner as the plots that received POM. The actual amount of POM applied was 720 g m^-2^ for target addition rates of 288 g C m^-2^. Because the ecosystem above and below-ground biomass production was 480 g C m^-2^ yr^-1^ (Ma, unpublished data), these POM additions correspond to increases in ecosystem biomass production of 60%. The soil organic C content before POM addition was 3.43 kg m^−2^ (SE = 0.13; n = 15) in the top 10 cm layer. The POM addition represented an increase in SOC of 8% in the top layer. However, due to the predominance of nonlabile C in most soils [46], POM addition in our study was expected to markedly enhance the labile C pool of the 0–10 cm soil layer.

For water addition treatments simulating a 30% increase in summer precipitation from 2010 to 2011, two open-top iron boxes (length 85 cm×width 71.5 cm×height 15 cm) were set outside each water addition plot. The base area of each iron box was approximately 15% of every plot (2×2 m). A circular hole (1.5cm inner diameter) was punched on one side of the box (facing the plot) and a rubber water pipe (1.5cm inner diameter) was connected to the hole. The rains fell into the boxes was rapidly transferred from the boxes to plots by these water pipes. Each pipe was an S-shaped distribution on the ground and many small holes were drilled along the pipe so that the rains uniformly flowed into the water addition treatment.

### Soil Samplings

Soil core samples were collected from the topsoil (0–10 cm) of all the plots in late June, mid-August and September in 2010 and 2011. Four cores (5 cm inner diameter, 10 cm length) were collected at each plot. The four replicates in each plot were pooled and mixed to get one composite sample and then brought immediately to the laboratory for analyses. The fresh samples were sieved using a 2 mm sieve and visible plant tissues were removed. Two subsamples of the sieved soil from each composite sample were obtained; one was kept in the refrigerator at 4°C for routine analyses and the other at −70°C, for phospholipid fatty acids (PLFAs) analysis.

### Soil Microclimate and Nutrient Measurements

Soil temperature and water content measurements were conducted one day after the rainfall events. Soil temperature at the depth of 10 cm was measured using a temperature probe connected to a Li-6400 (Li-Cor, USA). Gravimetric soil water content was measured by oven-drying samples at 105°C for 24 h. Concentrations of inorganic N (NH_4_^+^-N and NO_3_^-^-N) in the filtered extracts were determined using a flow injection autoanalyzer (FIAstar 5000 Analyzer, Foss Tecator, Denmark). Soil organic C and total N contents were measured by the dichromate oxidation method [47] and Kjeldahl method [48].

### Soil Microbial Biomass and Activity Measurements

Soil microbial biomass C and N were measured by fumigation-extraction method [49]. Briefly, the fresh soil samples were incubated for one week at 25°C after adjusting to 60% of water holding capacity in the dark. Then the moist samples (15 g dry weight equivalent) were fumigated for 24 h with CHCl_3_. Soil extracts from the fumigated and unfumigated samples were obtained by shaking soil samples with 60 ml 0.5 M K_2_SO_4_ for 30 min. The extracts were filtered through 0.45 µm filters and their extractable organic C and inorganic N analysed by dichromate digestion and Kjeldahl digestion as described by Lovell et al. [50]. Microbial biomass C and N were calculated as the difference in extractable organic C and inorganic N contents between the fumigated and the unfumigated samples using conversion factors (k_ec_ and k_en_) of 0.38 and 0.45 [50], respectively.

Soil microbial activity, i.e. the microbial respiration, was estimated by determining CO_2_ evolution over 2-wk incubation period. Respired CO_2_ was then captured in 5.0 ml of 0.5 M NaOH contained in a beaker suspended inside each Mason jar [36]. The NaOH solution was removed and titrated to determine the amount of CO_2_ evolved. The soil microbial activity was expressed as mg CO_2_ kg^-1^ day^-1^. The metabolic quotient (*q*CO_2_) was calculated as: [(mg CO_2_ C evolved in 14 days kg^-1^soil)/(mg microbial biomass C kg^-1^soil)/(14 days×24 h) ×1000] with the unit being mg CO_2_ C g^-1^C_mic_ h^-1^ [51].

### Microbial Community Structure

Phospholipid fatty acids (PLFAs) were extracted and quantified from 8.0 g (dry weight equivalent) soils using a procedure described by Bossio et al. [52]. The separation and identification of extracted PLFAs were carried out according to the standard protocol of the Sherlock Microbial Identification System V_4.5_ (MIDI) and a Gas Chromatograph (Agilent 6850, USA). Fatty acid nomenclature used in this study was as that defined by Bossio et al. [52]. The fatty acids i15: 0, a15: 0, i16: 1c, i16: 0, 16: 1ω7c, i17: 0, 17: 1ω6c, a17: 0, 17: 0cy, 18: 1ω7c, 18: 1ω5c and 19: 0cy were chosen to represent the PLFAs of the bacterial group [53], [54]. Three fatty acids (16: 1ω5c, 18: 2ω6, 9c and 18: 1ω9c) were chosen to represent the fungal group [54], [55]. Data from the PLFAs was presented as the percentage of the total PLFAs detected within a sample. Total percentages of PLFAs identified for each microbial group was calculated to represent their relative contributions to the total microbial biomass. The ratio of fungal to bacterial PLFAs was also included in the data analysis. This ratio has often been used as the indicator of the change in the soil microbial community structure [24], [56].

### Plant Biomass

In August 2010, one 1m ×1m quadrat was established in each plot. Presence of species in the measured quadrats was recorded as species richness of the plant community in August 2010 and 2011. Individual species frequency was used as the abundance of the species [57]. Plant species were categorized into two functional groups: grasses and forbs. Plant height of each species within a plot was the mean values of at least four random measurements of the species height.

We conducted a nondestructive method by developing regression equations to estimate peak plant species biomass in this study. In order to include all the species occurred in our study, we set 15 random calibration plots (1m×1m) near our experimental plots in both years. We also measured the specie frequency of each species, and then we clipped living aboveground biomass in the calibration plots and separated into difference species. Living plant aboveground tissues were separated from dead tissues, oven-dried at 65°C for 48 h, and weighed. We developed regression equations among peak biomass and specie frequency and plant height for each species for the calibration plots. All species showed good correlations between peak biomass and specie frequency and plant height in both years. Finally, we estimated the peak biomass of each species in the four treatments plots using the regression equations. Peak grasses and forbs biomass in each plot was the sum of biomass of grass and forbs species, respectively. On September 2010 and 2011, above-ground living tissues were harvested from one randomly located, 0.5m×0.5m quadrat of each plot, respectively, and root biomass was determined by soil coring to a depth of 10 cm using a cylindrical root sampler (9-cm inner diameter). Living and dead root fragments were separated based on color and consistency [58]. All samples of above-ground living tissues and roots were oven-dried at 65 °C to constant weight. Plant aboveground and root C and N contents measured by the dichromate oxidation method [47] and Kjeldahl method [48]. After analysis, plant samples were returned to their respective plots to maintain natural litter levels.

### Statistical Analysis

Seasonal mean values used in this study were calculated from the monthly mean values, which were first averaged from all measurements in the same month. Four-way ANOVAs were used to examine year (growing season), sampling times, soil carbon addition, water addition, and their interactions on soil microclimate, soil microbial properties, and inorganic N. Three-way ANOVAs were used to examine year, soil carbon addition, water addition, and their interactions on aboveground biomass, root biomass, grass and forb biomass, the ratio of grass to forb biomass, soil organic C, and soil total N content. Multiple comparisons were also performed to permit separation of effect means using the least significant difference test at a significance level of *P*<0.05. Correlation analyses were used to determine the relationships among soil microbial properties, soil microclimate, soil inorganic N, and soil organic C and total N pools. Data management and statistical analyses were performed using SPSS 11.5 software (SPSS, Chicago, IL, USA).
